# Peyer’s Patches and Mesenteric Lymph Nodes Cooperatively Promote Enteropathy in a Mouse Model of Food Allergy

**DOI:** 10.1371/journal.pone.0107492

**Published:** 2014-10-07

**Authors:** Haruyo Nakajima-Adachi, Akira Kikuchi, Yoko Fujimura, Kyoko Shibahara, Tsuyoshi Makino, Masae Goseki-Sone, Miran Kihara-Fujioka, Tomonori Nochi, Yosuke Kurashima, Osamu Igarashi, Masafumi Yamamoto, Jun Kunisawa, Masako Toda, Shuichi Kaminogawa, Ryuichiro Sato, Hiroshi Kiyono, Satoshi Hachimura

**Affiliations:** 1 Department of Applied Biological Chemistry, Graduate School of Agricultural and Life Sciences, The University of Tokyo, Tokyo, Japan; 2 Department of Microbiology and Immunology, Institute of Medical Science, The University of Tokyo, Tokyo, Japan; 3 Biotechnical Center (BT Center), Japan SLC, Inc., Shizuoka, Japan; 4 Department of Food and Nutrition, Faculty of Human Sciences and Design, Japan Women’s University, Tokyo, Japan; 5 Core Research for Evolutional Science and Technology (CREST), Japan Science and Technology Agency, Tokyo, Japan; 6 Department of Microbiology and Immunology, Nihon University School of Dentistry at Matsudo, Chiba, Japan; 7 International Research and Development Center for Mucosal Vaccines, Institute of Medical Science, The University of Tokyo, Tokyo, Japan; 8 Laboratory of Vaccine Materials, National Institute of Biomedical Innovation, Osaka, Japan; 9 Vice President’s Research Group “Molecular Allergology”, Paul-Ehrlich-Institut, Langen, Germany; 10 Department of Food Bioscience and Biotechnology, Nihon University, Kanagawa, Japan; 11 Department of Medical Genome Science, Graduate School of Frontier Science, The University of Tokyo, Tokyo, Japan; 12 Graduate School of Medicine, The University of Tokyo, Tokyo, Japan; 13 Research Center for Food Safety, Graduate School of Agricultural and Life Sciences, The University of Tokyo, Tokyo, Japan; Fox Chase Cancer Center, United States of America

## Abstract

**Background and Objective:**

To improve the efficacy and safety of tolerance induction for food allergies, identifying the tissues responsible for inducing intestinal inflammation and subsequent oral tolerance is important. We used OVA23-3 mice, which express an ovalbumin-specific T-cell receptor, to elucidate the roles of local and systemic immune tissues in intestinal inflammation.

**Methods and Results:**

OVA23-3 mice developed marked enteropathy after consuming a diet containing egg white (EW diet) for 10 days but overcame the enteropathy (despite continued moderate inflammation) after receiving EW diet for a total of 28 days. Injecting mice with anti-IL-4 antibody or cyclosporine A confirmed the involvement of Th2 cells in the development of the enteropathy. To assess the individual contributions of Peyer’s patches (PPs), mesenteric lymph nodes (MLNs), and the spleen to the generation of effector CD4^+^ T-cells, we analyzed the IL-4 production, proliferation in response to ovalbumin, and CD4^+^ T-cell numbers of these tissues. EW feeding for 10 days induced significant IL-4 production in PPs, the infiltration of numerous CD4^+^ T-cells into MLNs, and a decrease in CD4^+^ T-cell numbers in spleen. On day 28, CD4^+^ T-cells from all tissues had attenuated responses to ovalbumin, suggesting tolerance acquisition, although MLN CD4^+^ T-cells still maintained IL-4 production with proliferation. In addition, removal of MLNs but not the spleen decreased the severity of enteropathy and PP-disrupted mice showed delayed onset of EW-induced inflammatory responses. Disruption of peripheral lymphoid tissues or of both PPs and MLNs almost completely prevented the enteropathy.

**Conclusions:**

PPs and MLNs coordinately promote enteropathy by generating effector T-cells during the initial and exacerbated phases, respectively; the spleen is dispensable for enteropathy and shows tolerogenic responses throughout EW-feeding. The regulation of PPs may suppress the initiation of intestinal inflammation, subsequently restricting MLNs and inhibiting the progression of food-allergic enteropathy.

## Introduction

The prevalence of food allergies is increasing, particularly in westernized countries [1]. Among the prospective allergen-specific treatments for food allergies that have been investigated in current clinical practice, specific oral tolerance immunotherapy has attracted considerable interest [2]. However, several reports suggest that this immunotherapy can provoke severe adverse reactions (e.g. gastrointestinal inflammation and anaphylaxis) to the allergenic diet necessary to induce specific oral tolerance. For specific oral tolerance immunotherapy to be safe and effective, the mechanisms underlying the induction of food allergies and subsequent development of oral tolerance must be elucidated in detail, including identification of the tissue involved, so that targeted therapy with decreased risk to patients can be developed [3], [4].

The intestinal immune tissue is a primary site of sensitization to food allergens. Gut-associated lymphoid tissues (GALT) may play an important role in causing food allergic intestinal inflammation and subsequent induction of oral tolerance [5], [6]. GALT comprises both immune-inductive sites–Peyer’s patches (PPs), isolated lymphoid follicles, and mesenteric lymph nodes (MLNs)–and effector sites, which include the lamina propria and intestinal epithelium. How GALT itself responds to food allergens, the relationship between the intestinal and systemic (e.g., splenic) responses to food allergens disseminated from GALT, and the role this association plays in determining tolerance versus inflammation remain unclear as yet. Previous studies revealed that effector cells from spleen, a key systemic immune tissue [7], or MLN [8] or PP cells [9] caused gastrointestinal inflammation. These results suggest both GALT and spleen act as effector tissues, but how these tissues function–both independently and cooperatively–has not been elucidated clearly [6].

Traditional food allergy mouse models involve primary sensitization with a food antigen and adjuvant, followed by oral administration of the antigen. However, this process fundamentally alters the immune responses of the mice [10], complicates direct analysis of the antigen-specific local or systemic immune responses that are triggered by orally administered antigen, and complicates efforts to elucidate the mechanisms of food allergy. Detailed analysis of the mechanisms underlying food-allergic intestinal inflammation and subsequent oral tolerance requires an improved mouse model.

In our previous study, we established a model of food allergy by using OVA23-3 mice, a transgenic line that expresses a T-cell receptor specific for ovalbumin (OVA; a leading egg allergen) [11], [12]. Disproportionate Th2-skewed responses and food-allergic enteropathy with weight loss can be induced in OVA23-3 mice simply by feeding them an egg-white (EW)-based diet [12], [13]. Furthermore, continued feeding of the EW diet is accompanied by reversal of extant inflammation. This enteropathy is similar to food protein-induced enteropathy of infancy [14]. Compared with traditional mice models, the OVA23-3 model is characteristic in manifesting the enteropathy by feeding diet alone, whereby we can follow the immune responses initiated from feeding diet in the absence of extraneous and potentially confounding effects due to adjuvant. To identify the key tissues in both food-allergic enteropathy and subsequent oral tolerance, we here examined the responses of CD4^+^ T-cells purified from PPs, MLN, and spleen of intact EW-diet-fed OVA23-3 mice and of those from which we removed various lymphoid tissues. Our data revealed that both the MLNs and PPs of GALT are integral to the development of food-allergic intestinal inflammation through their cooperative roles in generating and maintaining IL-4-producing CD4^+^ T-cells. In addition, we learned that the spleen contributes to the induction of systemic tolerance to EW diet in OVA23-3 mice, but is dispensable for the development of enteropathy. In particular, our chronologic analysis clearly showed that PPs are an important tissue as an accessible target in inducing oral tolerance to inhibit subsequent aggravation of the inflammatory responses in MLNs.

## Materials and Methods

### Ethics statements

We followed the ARRIVE Guidelines for preparing current experiments and submitting this manuscript. The all aspects of current experiments including anesthesia, euthanasia and the withholding of perioperative analgesia were approved by the Animal Use Committee of the Faculty of Agriculture at The University of Tokyo and we followed the guidelines of The University of Tokyo for the care and use of animals (approval no. P11-533). During surgery, mice were anesthetized by isoflurane, which was performed under aseptic condition. Perioperative analgesia was withheld, due to its pharmacological effects on inducing enteropathy and maintaining intestinal homeostasis, which was a primary parameter evaluated in the study [15∼18]. When necessary for cellular and histological analysis, mice were euthanized by cervical dislocation by experts. Although the mice developed weight loss and enteropathy by day 10 of feeding the egg-white diet, they did not exhibit any other clinical signs (e.g., hunched body position, ruffled fur, decreased mobility/activity, severe weight loss) and recovered from the inflammation upon continued feeding of the diet for a total of 28 days. Therefore, our experiments without setting preemptive humane endpoints were approved.

### Mice

OVA23-3 mice were generously provided by S. Habu (Tokai University School of Medicine, Japan) [11]. Heterozygous OVA23-3 mice (obtained by backcrossing homozygous OVA23-3 mice with BALB/cA mice [CLEA Japan, Tokyo, Japan]) were maintained under specific pathogen-free and controlled conditions at The University of Tokyo (sterilized deionized drinking water; sterilized commercial chow; room temperature; 22°C, 12∶12-h light:dark cycle). These conditions were monitored daily. Each cage (182×260×128 mm, [catalog no. CL-0103-2, CLEA Japan, Tokyo, Japan]) contained 5 or fewer mice.

### Generation of OVA23-3 mice devoid of PPs or peripheral lymphoid tissue (pLT)

To generate OVA23-3 mice devoid of PPs (PP^–^ mice), female BALB/c mice were bred with homozygous OVA23-3 mice; on gestational day 14.5, the female mice were injected intravenously with 1 mg of anti-IL-7Ralpha monoclonal antibody (mAb; A7R34, generated and donated by S. Nishikawa [Kyoto University, Japan]) [19]. The frequencies of CD4^+^ and CD8^+^ T-cells in thymic, splenic, intestinal epithelial and mesenteric lymphocytes were comparable between PP-normal and PP-deficient of BALB/c or OVA23-3 mice (data not shown). To generate OVA23-3 mice devoid of pLT (pLT^–^ mice), female BALB/c mice were bred with homozygous OVA23-3 mice; on gestational days 11, 14, and 17, female mice were injected intravenously with 200 microgram each of LTbetaR-Ig and TNFR55-Ig fusion proteins (these regents were gifts from P. D. Rennert [Biogen Idec, Inc., Cambridge, MA]) [20], [21]. The disruption of PP or pLT organogenesis in the offspring was confirmed by observation under a stereomicroscope (Leica Microsystems Japan, Tokyo, Japan) or by intravenously injecting 200 microliter of 1% Evans blue in PBS into EW-fed OVA23-3 mice to visualize lymph nodes.

### Surgery

The organogenesis of MLNs or spleen cannot be disrupted by injection of mAb or fusion proteins into pregnant mice because specialized differentiation pathways of these tissues remain unclear. Therefore, we used surgery so that we might define the roles of the individual tissues. Mesenteric lymphadenectomy [22] and splenectomy of OVA23-3 mice (age, 6 to 7 weeks old) was performed in collaboration with Japan SLC (Hamamatsu, Japan). Mice were used in experiments once they had regained their postsurgical weight loss, 7 to 10 days after from the surgery. During surgery, mice were anesthetized by isoflurane, which was performed under aseptic condition, and abdominal tissues were bathed with sterile warm (37°C) PBS to prevent drying, and mice recovered from anesthesia and surgery in a warmed (40°C) clean cage. After surgery, mice were monitored (weight and activity) and the incision was cleaned daily. The completeness of lymphadenectomy was confirmed by observation under a stereomicroscope (Leica Microsystems Japan, Tokyo, Japan) or by intravenously injecting 200 microliter of 1% Evans blue in PBS into EW-fed OVA23-3 mice to visualize lymph nodes.

### EW diet and OVA uptake

For 28 d, male OVA23-3 mice (age, 6 to 8 weeks) were fed experimental solid diets in which the 20% protein fraction comprised EW (EW diet) or casein (CN; control diet) [12]. Mice were allocated to each group so that average weight did not differ significantly between groups and were weighted daily through day 10 and then every 4 to 7 days until the end of the 28-day experimental period. When necessary for cellular and histological analyses, mice were weighed, euthanized, and their tissues removed for further analysis. Sera were stored at −80°C. Serum OVA-specific IgE levels were analyzed by ELISA as described previously [12] and measurement of the serum concentration of OVA (OVA uptake) is described in File S1.

### Treatment with cyclosporine A and anti-IL-4 mAb

Cyclosporine A (Astellas Pharma, Tokyo, Japan) was dissolved (final concentration, 1 µg/µL) in elution buffer (0.05% Tween-80 and 10% ethanol in 0.9% NaCl) and administered intraperitoneally every other day throughout the 10-day experimental period. Anti-IL-4 mAb (1 mg; clone 11B11) [23] or control Ab (1 mg; rat IgG, Cappel, Cochranville, PA, USA) was administered intravenously on the day before and on day 7 of feeding the diet.

### Intestinal histology

Segments (3 cm each) of jejunum (just after or 8 cm distal to the duodenum) were harvested, opened longitudinally, fixed with 10% formalin, and stained with hematoxylin and eosin for morphologic evaluation [12].

### Measurement of alkaline phosphatase activity

The 1-cm section of jejunum that lay 7 cm distal to the duodenum was harvested, opened longitudinally, and washed with sterile cold PBS. Using 2 glass slides, we scraped the intestinal mucosa from these segments and stored it at –80°C prior to homogenization. The mucosa were homogenized in 10 mM Tris-buffered saline (pH 7.3) containing 1% Triton X-100 and 1 mM phenylmethylsulfonylfluoride and then centrifuged at 7,000×*g* for 15 min. Alkaline phosphatase activity in the supernatants of the intestinal tissue homogenates was analyzed by using an enzyme substrate (10 mM *p*-nitrophenyl phosphate in 100 mM 2-amino-2-methyl-1, 3-propanediole HCL buffer containing 5 mM MgCl_2_; pH 10.0) at 37°C [24]. Enzyme activity was defined as the rate of hydrolysis of *p*-nitrophenyl phosphate and expressed in units (1 U = 1 µmol *p*-nitrophenol formed in 1 min). The protein concentration of each sample was standardized to 1 mg by using BCA protein assay reagent (Pierce, Rockford, IL, USA).

### Assessment of CD4^+^ T-cell functions

CD4^+^ T-cells were prepared from MLNs, PPs, and spleen, and their proliferation, cytokine production, and infiltration into various tissues were evaluated according to procedures provided in File S1 [12]. Protocols for isolating small intestinal lymphocytes and for measuring CD4^+^ T-cell number and rate in small intestinal lymphocytes [25] are provided also in File S1.

### Statistical analysis

Results are presented as mean ± 1 SD. The Mann–Whitney *U* test, Steel, and Shirley–Williams tests were used for nonparametric analysis. Differences were considered significant when the *P* value was less than 0.05 or 0.01, depending on the test.

## Results

### IL-4-producing CD4^+^ T-cells were indispensable for the clinical manifestation of food allergy in OVA23-3 mice

**Figure 1:**
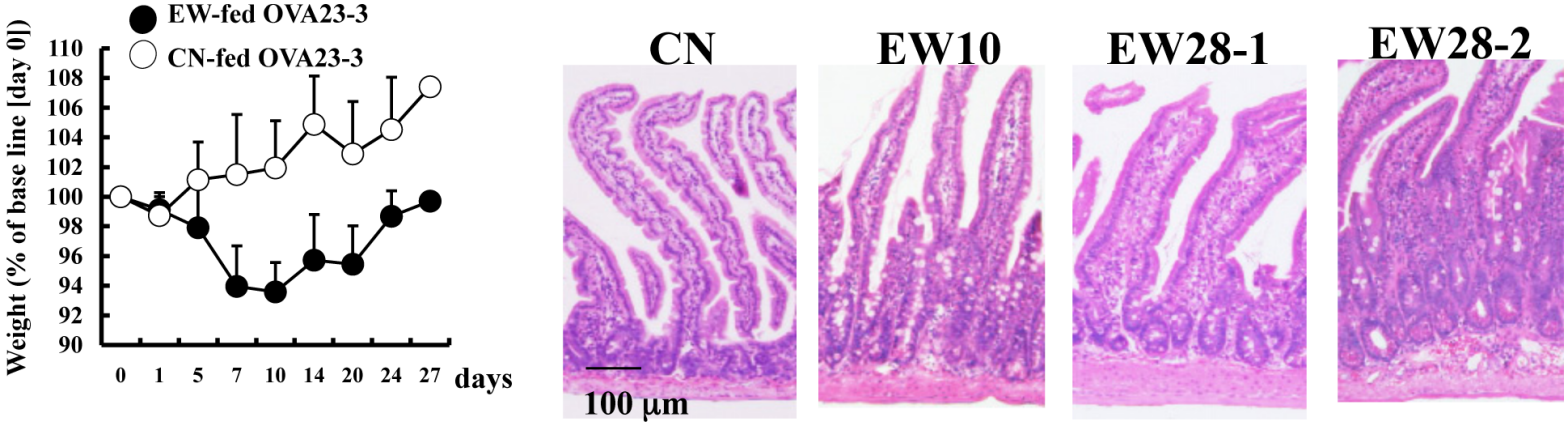
An OVA-specific mouse model of food allergy demonstrates enteropathy followed by oral tolerance. Weight changes and jejunal sections (hematoxylin and eosin stain) throughout EW-diet feeding of OVA23-3 mice (open circles: CN-fed, n = 5; solid circles: EW-fed, n = 7). The data shown are representative of four independent experiments.

As described in our previous study [12], OVA23-3 mice demonstrated marked enteropathy, with weight loss (i.e., <10% of baseline body weight), from day 4 until day 7 to 10 after they began receiving the EW diet. Although particularly apparent in the jejunum, inflammation occurred throughout the small intestine and manifested as enteropathy involving goblet-cell hyperplasia, crypt elongation, villous atrophy and inflammatory cell infiltration (Figure 1). As the mice continued to receive the EW diet until day 28, they regained body weight and demonstrated repair of injured intestinal tissues, although moderate inflammation remained. As indicated previously [12], the cells that infiltrated into the small intestine were eosinophils, mast cells, neutrophils, macrophages, and CD4^+^ T-cells (Figure S1 and Result S1). Excess CD4^+^ T-cells function may contribute to the infiltration of other cells. Serum OVA-specific IgE responses were increased (*P*<0.01) on day 21 compared with the level before receiving EW diet. On day 28, the IgE responses were significantly (*P*<0.05) decreased compared with the level on day 21, but still elevated higher (*P*<0.05) than the level before receiving EW diet. (Figure S2 and Result S2).

We previously showed that EW-fed RAG-2 deficient OVA23-3 mice, in which nearly 100% of CD4^+^ T-cells are OVA-specific, developed a similar enteropathy to that of the OVA23-3 mice in the current study [12]. Therefore, our results indicate that OVA-specific CD4^+^ T-cells are involved in the induction and resolution of weight loss and intestinal inflammation in this model, because more than 90% of the peripheral CD4^+^ T cells in OVA23-3 mice are OVA-specific [11].

**Figure 2:**
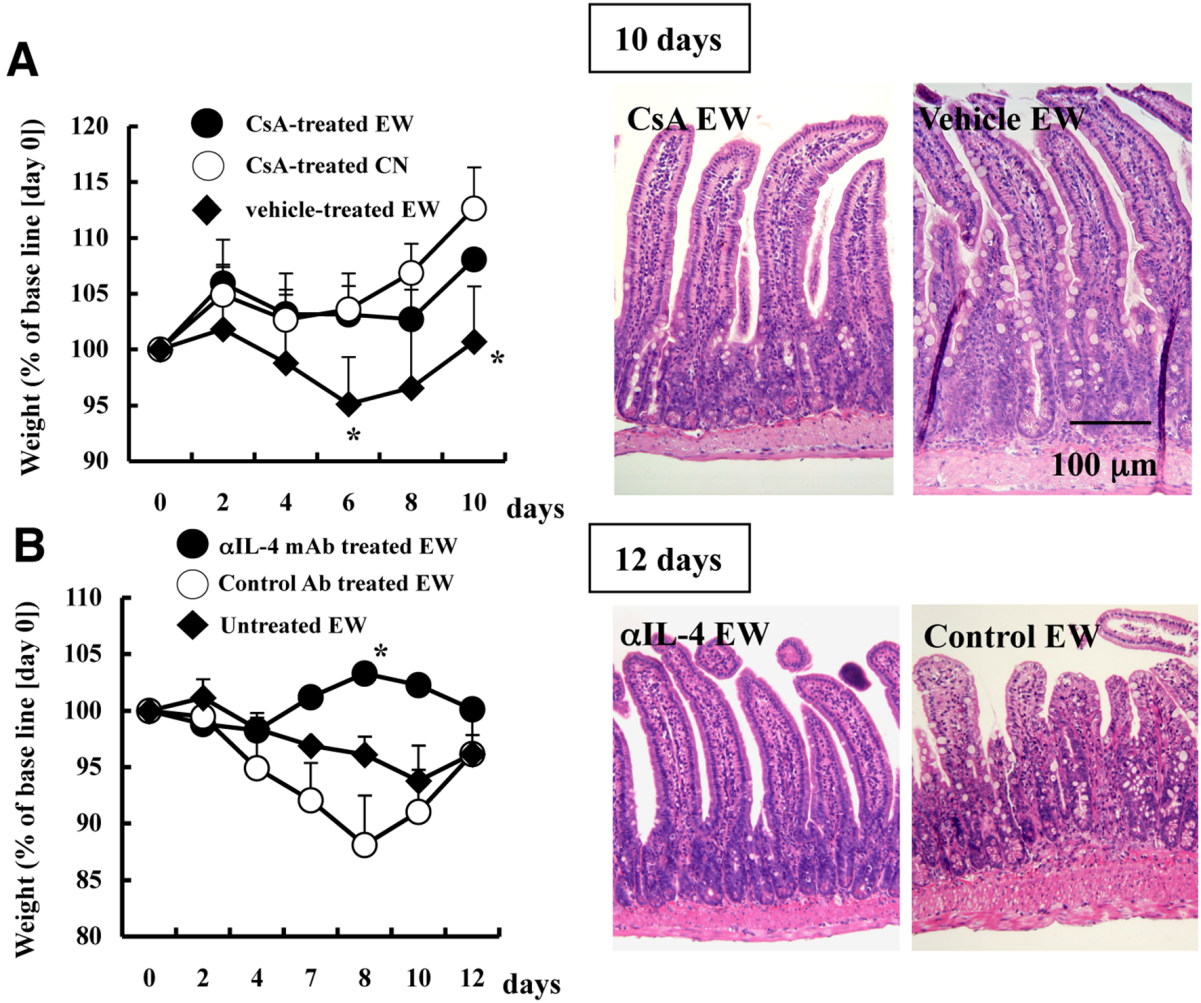
Roles of T-cells and IL-4 in food-allergic intestinal inflammation. Weight changes and jejunal sections (hematoxylin and eosin stain) of EW- or CN-fed OVA23-3 mice treated with (A) cyclosporine A (CsA) or (B) anti-IL-4 mAb. Morphologic changes were analyzed between days 10 and 12. (A) Open circles: CsA-treated, CN-fed (CN, n = 3); solid circles: CsA-treated, EW-fed (EW, n = 5); solid diamonds: vehicle (control)-treated EW-fed (n = 4). (B) Open circles: Control Ab-treated (EW, n = 3); solid circles: anti IL-4-treated (EW, n = 3); solid diamonds, untreated (EW, n = 2). *, Value is significantly (*P*<0.05) different from that for vehicle-treated EW or control Ab-treated EW mice. All data are representative of three independent experiments.

In our previous study, the clinical manifestation of EW-fed OVA23-3 mice was associated with Th2 CD4^+^ T-cell responses [12]. Other studies have used IL-4-deficient mice to illustrate the role of IL-4 in food allergic diarrhea [26], [27] or aversion to oral administered OVA [28]. To verify the importance of Th2 cells in our model, we treated OVA23-3 mice with cyclosporine A, an inhibitor of T-cell activation, or anti-IL-4 mAb. Both treatments markedly decreased the expected weight loss and intestinal inflammation in EW-fed OVA23-3 mice (Figure 2A and 2B). These results suggest that IL-4-producing CD4^+^ T-cells are indispensable effector cells in the induction of disease symptoms of EW-fed OVA23-3 mice.

### MLN CD4^+^ T-cells, but not PP or splenic CD4^+^ T-cells, maintained IL-4 production during feeding of the EW diet

To analyze the contribution of the primary inductive sites of GALT and a tissue central to systemic immunity to the generation of effector T cells, we examined CD4^+^ T-cell function–specifically, cell number, proliferation and IL-4 production–in the MLNs, PPs, and spleen of OVA23-3 mice after stimulation with OVA. These functions were assessed before the induction of inflammation (that is, during feeding of the CN diet) and during the marked (days 7 through 10 of feeding the EW diet) and moderate (day 28 of the EW diet) phases of inflammation.

**Figure 3:**
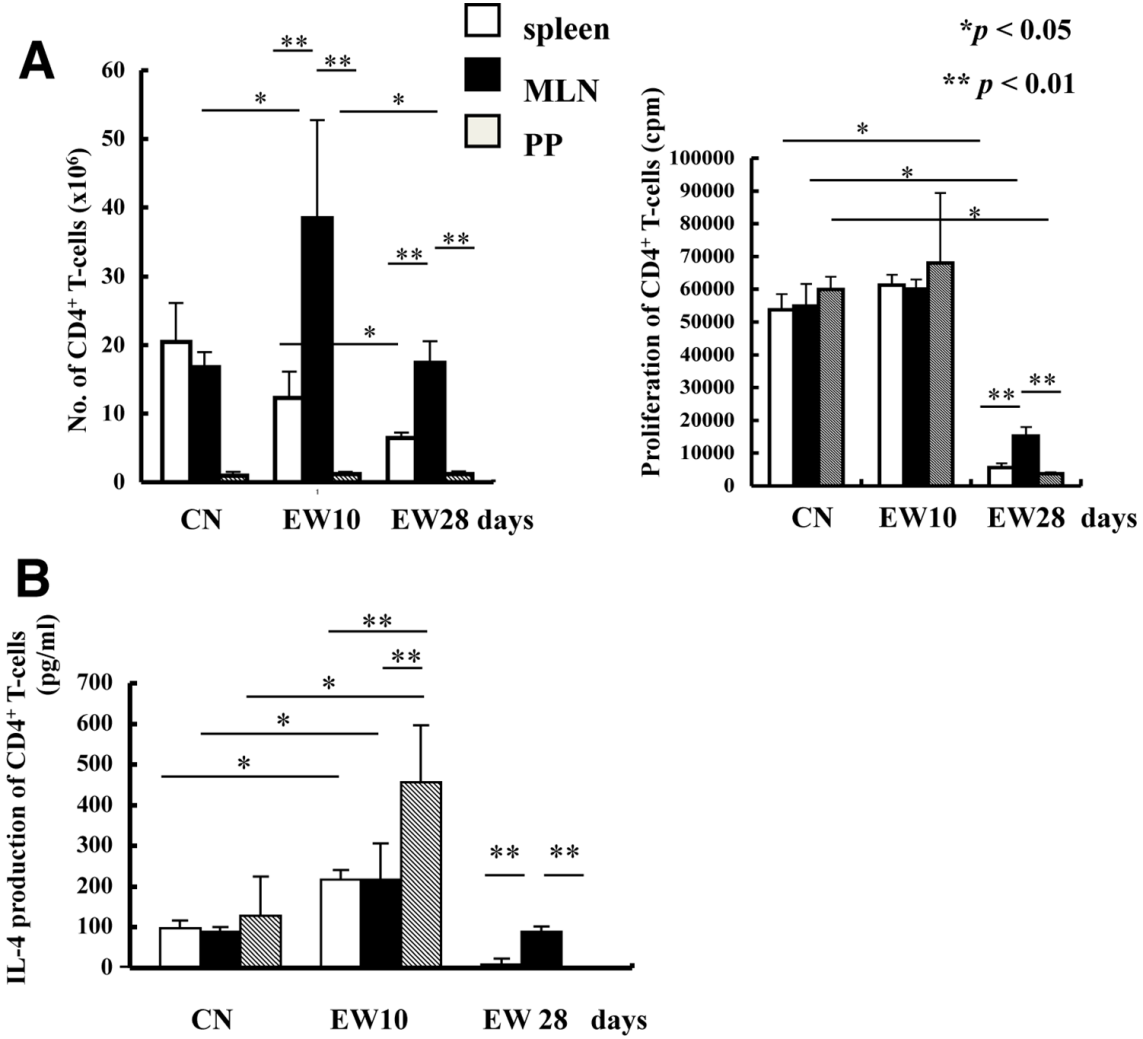
Roles of CD4^+^ T-cells from PPs, MLNs, and spleen differ in EW-fed OVA23-3 mice. (A) Number of CD4^+^ T-cells (mean±1 SD) that infiltrated into (left, *n* = 3) and the proliferation of CD4^+^ T-cells (mean±1 SD) purified from (right, *n* = 4) the PPs, MLNs, and spleens of OVA23-3 mice on the EW or the CN diet over time. (B) IL-4 production (mean±1 SD) by CD4^+^ T cells from each tissue of OVA23-3 mice (*n* = 4) on the EW or the CN diet over time. All data are representative of two independent experiments.

The number of splenic CD4^+^ T-cells was decreased on day 10 (*P*<0.1) and 28 (*P*<0.05) in EW-diet-fed mice compared with the CN-diet group. In contrast, the number of CD4^+^ T-cells in PPs remained consistently low throughout EW-diet feeding, whereas that in MLNs increased significantly (*P*<0.05) on day 10 compared with cell counts in CN-fed mice and decreased (*P*<0.05) on day 28 from that on day 10 of EW diet. The number of CD4^+^ T-cells in MLNs increased significantly (*P*<0.01) compared with those in spleen and PPs on day 10 and remained elevated (*P*<0.01) on day 28 of the diet (Figure 3A, left panel).

The proliferation of CD4^+^ T-cells from the PPs, MLNs, and spleen on day 10 was similar between CN-fed and EW-fed OVA23-3 mice. However, on day 28, CD4^+^ T-cell proliferation was significantly (*P*<0.05) lower in EW-fed compared with CN-fed mice. However, CD4^+^ T-cells from MLNs maintained the highest (*P*<0.01) level of proliferation among the 3 lymphoid tissues evaluated (Figure 3A, right panel). These results suggest a central role for MLNs, but not spleen or PPs in the intestinal inflammation.

IL-4 production by CD4^+^ T-cells from EW-fed OVA23-3 mice on day 10 was increased (*P*<0.05) compared with that in CN-diet groups in all 3 tissues; the difference was particularly apparent in PP CD4^+^ T-cells (*P*<0.01 compared with MLNs and spleen on day 10, Figure 3B). This finding suggests that, compared with those from MLNs or spleen, CD4^+^ T-cells from PPs play an important role in early inflammatory responses. On day 28, IL-4 production in CD4^+^ T-cells from spleen and PPs was dramatically lower (P<0.01) than that in CD4^+^ T-cells from MLNs, suggesting persistent role for MLNs in the inflammatory responses.

These results suggest that 1) the establishment of the enteropathy was dependent on the generation of IL-4-producing CD4^+^ T-cells in GALT; 2) long-term feeding of EW diet induced CD4^+^ T-cell tolerance through inhibition of IL-4 production in an OVA-specific manner; and 3) the induction of T-cell tolerance was followed by attenuation of intestinal inflammation and weight loss in EW-fed OVA23-3 mice.

### MLNs are required for the induction of intestinal inflammation in a mouse model of food allergy

**Figure 4:**
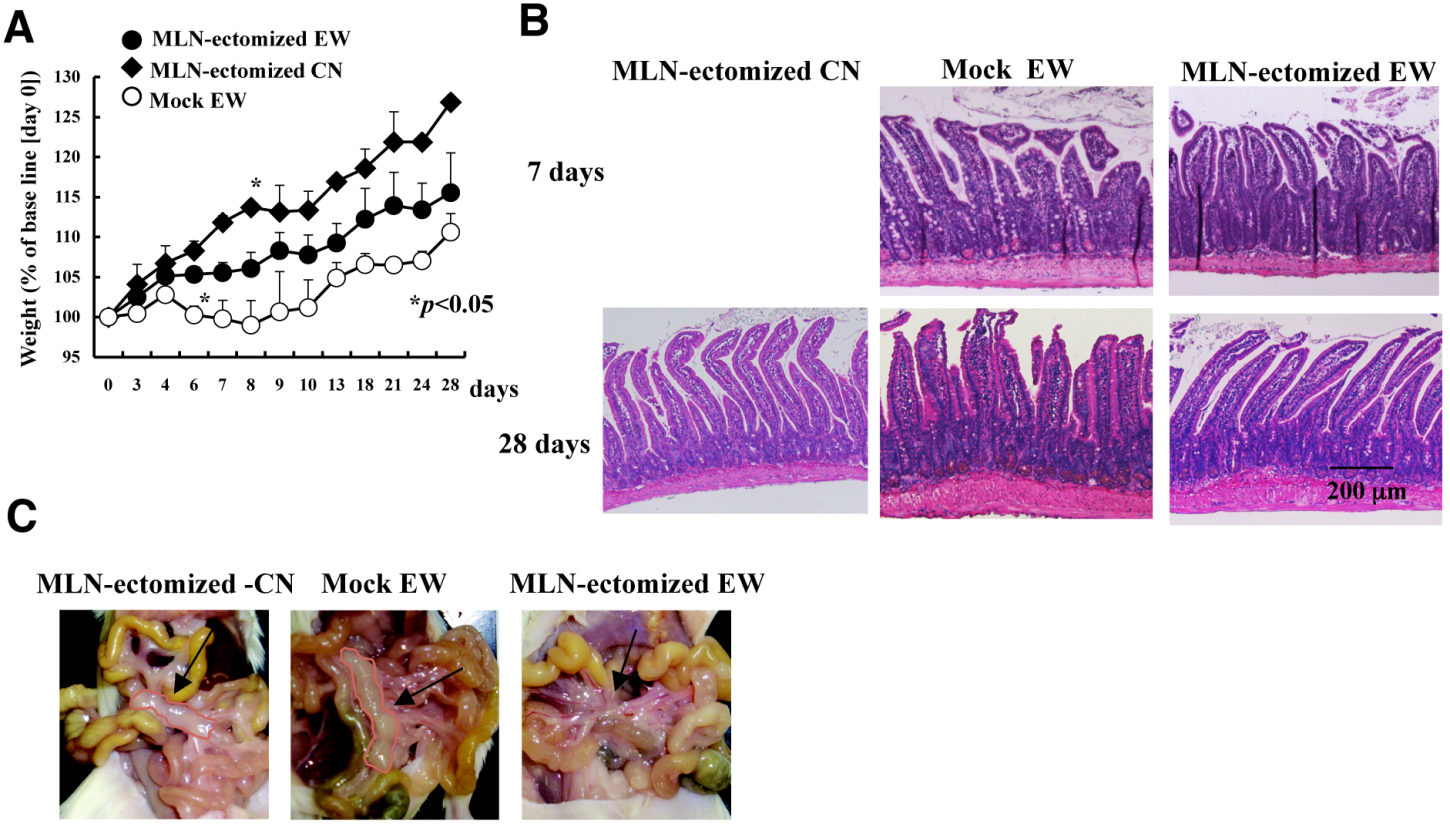
Potential contribution of MLNs to the intestinal inflammatory immune response. (A) Weight changes of MLN-ectomized EW-fed (EW, n = 7); sham-operated EW-fed (Mock, n = 4); and MLN-ectomized CN-fed (CN, n = 3) mice. *, Value is significantly (*P*<0.05) different between those for MLN-ectomized EW and sham-operated EW groups or those for MLN-ectomized EW and MLN-ectomized CN groups. (B) Jejunal sections obtained on day 7 (during inflammation, upper panels) or on day 28 (during tolerance, lower panels). (C) Arrows indicate MLNs or site of mesenteric lymphadenectomy in OVA23-3 mice on day 7 of the EW (Mock EW or MLN-ectomized EW) or CN (MLN-ectomized CN) diet. All data are representative of two independent experiments.

The inflammatory CD4^+^ T-cell functions in GALT and the maintenance of persistent CD4^+^ T-cell responses to dietary OVA in MLNs suggest that MLNs promote the development of enteropathy in OVA23-3 mice during EW-diet feeding. To examine this potential role, we surgically removed the MLNs from OVA23-3 mice and then fed the EW diet to the lymphadenectomized mice. Notably, MLN-ectomized EW-fed OVA23-3 mice showed significantly (*P*<0.05) less weight loss than did sham-operated EW-fed mice (Figure 4A). Moreover, compared with sham-operated mice, MLN-ectomized EW-fed OVA23-3 mice had milder intestinal inflammation, that is, only crypt hyperplasia on day 7 (Figure 4B, upper) and less cell infiltration on day 28 (Figure 4B, lower). The EW diet remarkably increased the size of the MLNs in sham-operated OVA23-3 mice (Figure 4C, center panel). In contrast, MLNs were not visible in MLN-ectomized EW-fed OVA23-3 mice, indicating that the tissues had been removed successfully through lymphadenectomy (Figure 4C, right panel). The swelling of the MLNs during severe inflammation in the EW-fed OVA23-3 mice may relate to the significant (*P*<0.01) increase in the number of CD4^+^ T-cells in MLNs on day 10 compared with those of spleen and PPs (Figure 3A). These results indicate that MLNs are indispensable for the induction of marked weight loss and intestinal inflammation in this mouse model of food allergy.

### PPs initiate the induction of intestinal inflammation in food-allergic mice

**Figure 5:**
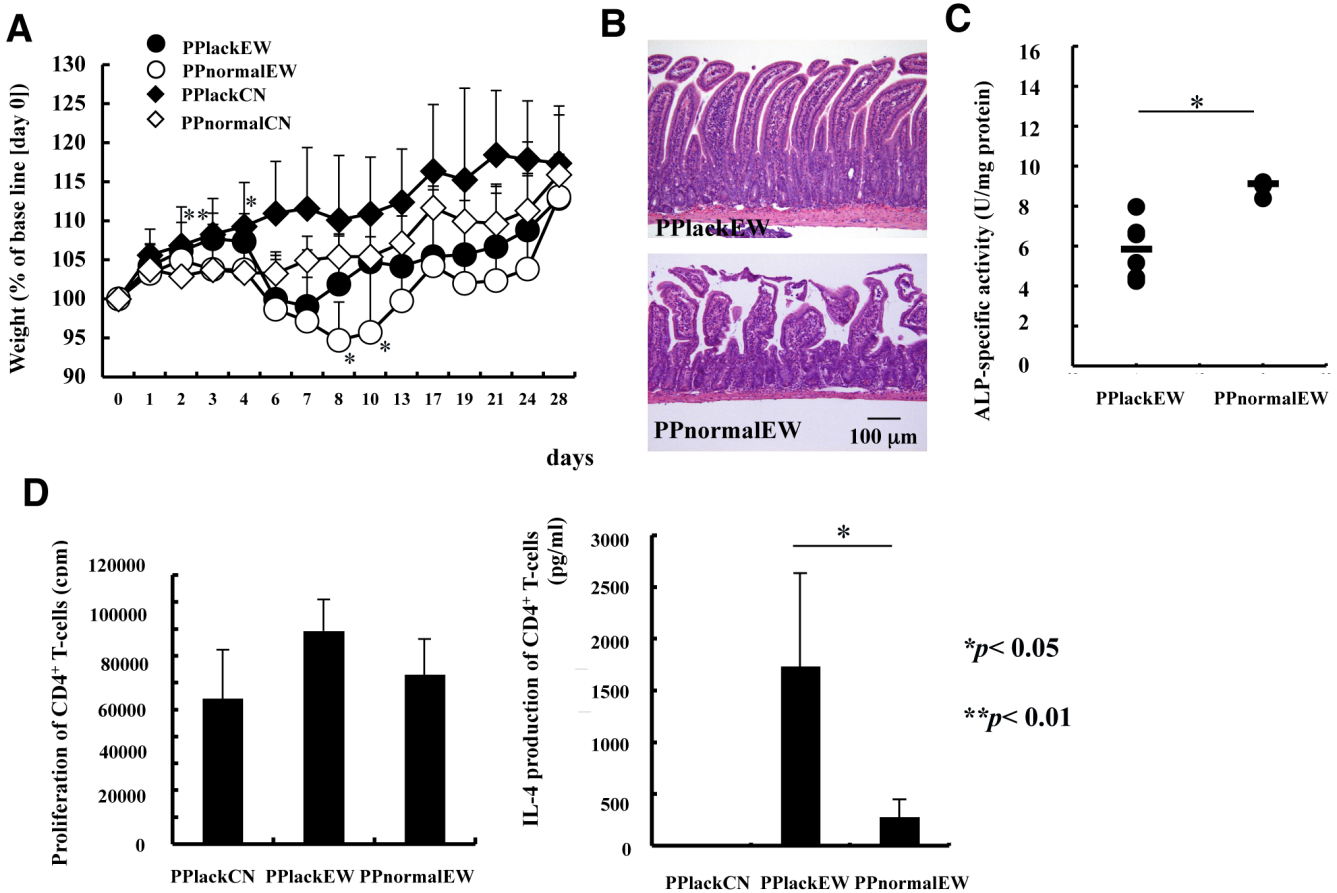
Manifestation of food allergy in EW-fed PP^–^ OVA23-3 mice. (A) Weight change in EW-fed PP^–^ (PPlackEW, n = 18); EW-fed PP^+^, (PPnormalEW, n = 17; CN-fed PP^–^ (PPlackCN, n = 8); and CN-fed PP^+^ (PPnormalCN, n = 8) mice. Value significantly (*, *P*<0.05; **, *P*<0.01) different between those for PP^–^ and PP^+^ EW-fed OVA23-3 mice. (B) Jejunal sections. (C) alkaline phosphatase activity in the jejunum (PPlackEW, n = 6; PPnormalEW, n = 3). (D) Proliferation of (left panel) and IL-4 production by (right panel) MLN CD4^+^ T-cells from PP^–^ or PP^+^ EW-fed OVA23-3 mice on day 3. Data are representative of two or three experiments.

The high levels of IL-4 production of PP CD4^+^ T-cells on day 10 of the EW diet and the moderate intestinal inflammation found in the MLN-ectomized mice suggest that PPs play a role in establishing intestinal inflammation in EW-fed OVA23-3 mice. To this end, we generated PP^–^ OVA23-3 mice by disrupting PP organogenesis, and fed them the EW diet. PP^–^ EW-fed OVA23-3 mice began to demonstrate weight loss on day 5 or 6, markedly later than did PP^+^ (normal) EW-fed OVA23-3 mice, which showed onset of weight loss on day 3 or 4. The normal growth of both CN-fed PP^–^ and PP^+^ OVA23-3 mice showed that weight loss was specific to the EW diet and was dependent on the presence of PPs. PP^–^ OVA23-3 mice began to recover from their weight loss on day 8 of the EW diet, which was earlier than did PP^+^ OVA23-3 mice (day 10; Figure 5A). This result suggests that PPs, through their role in the initial induction of inflammatory responses after orally administered OVA, delayed the recovery from weight loss in PP^+^ OVA23-3 mice.

To verify that the initial induction of the enteropathy occurred in PPs, inflammatory responses in the jejunum on day 3 or 4 of the EW diet were examined. PP^+^ but not PP^–^ OVA23-3 mice showed morphologic changes, including cell infiltration, villous atrophy, and an increased number of goblet cells (Figure 5B). In addition, alkaline phosphatase activity, an indication of inflammation [29], was significantly (*P*<0.05) lower in PP^–^ EW-fed than PP^+^ EW-fed OVA23-3 mice (Figure 5C). We then assessed the influence of PP disruption on CD4^+^ T-cell function in MLNs. On day 3 of the EW diet, MLN CD4^+^ T-cells from PP^–^ EW-fed OVA23-3 mice showed greater proliferation (*P*<0.1) (Figure 5D, left panel) and significantly (*P*<0.05) enhanced IL-4 production compared with those from PP^+^ EW-fed mice (Figure 5D, right panel). PP disruption did not alter OVA uptake by epithelial cells or its transport into sera (Figure S3 and Result S3). These results suggest that the delayed onset of inflammation in PP^–^ OVA23-3 mice reflects the lack of an immediate response of PP CD4^+^ T-cells against OVA; aggravation of the enteropathy likely developed only after antigen and antigen-presenting cells reached the MLNs to initiate the activation of and IL-4 production by MLN CD4^+^ T-cells and their migration into the lamina propria. In addition, these findings show that PPs are involved early in induction of enteropathy in this mouse model of food allergy.

### Coordinated contribution from PPs and MLNs is crucial to the development of enteropathy in a mouse model of food allergy

**Figure 6:**
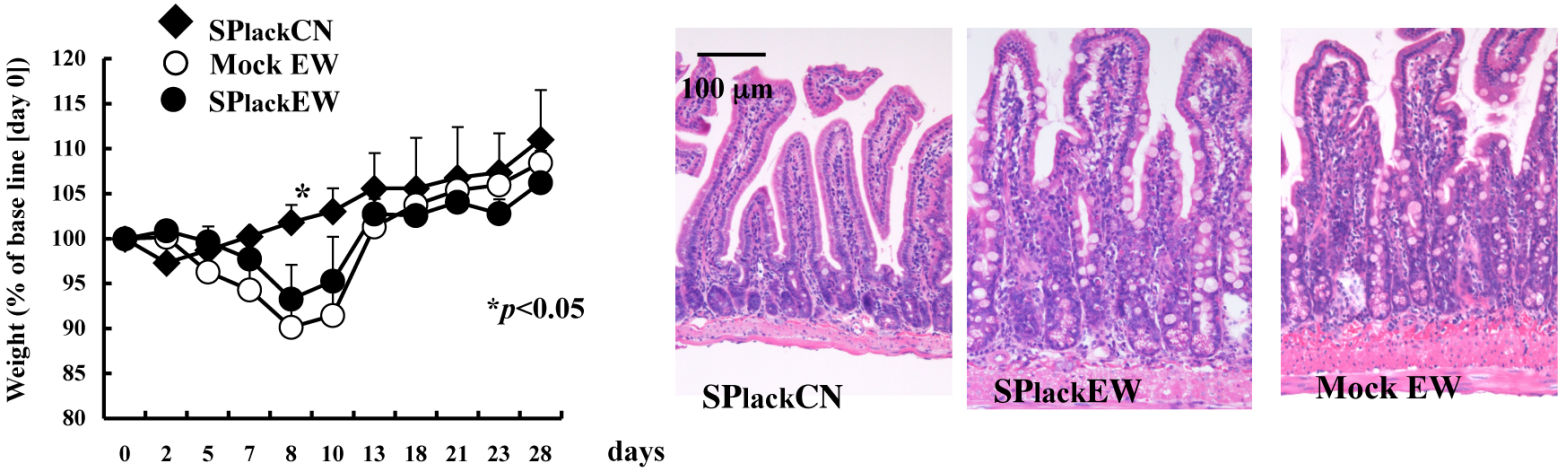
The spleen is dispensable for the establishment of enteropathy in EW-fed OVA23-3 mice. Weight change (left panel) and jejunal sections (right panel, day 7) of splenectomized EW-fed (SPlackEW, n = 5), splenectomized CN-fed (SPlackCN, n = 3), and sham-operated EW-fed (Mock EW, n = 4) OVA23-3 mice. *, Significant (*P*<0.05) difference between values for splenectomized CN-fed and EW-fed mice.

Neither disruption of PP organogenesis nor mesenteric lymphadenectomy alone completely abolished the EW-diet induced intestinal inflammation in OVA23-3 mice. To determine whether PPs and MLNs cooperate to induce enteropathy, we generated OVA23-3 mice devoid of both PPs and MLNs (PP^–^ MLN-ectomized mice). When fed the EW diet, PP^–^ MLN-ectomized OVA23-3 mice showed no associated weight loss or morphologic changes in the small intestine. In particular, the small intestinal villi of the EW-fed PP^–^ MLN-ectomized OVA23-3 mice were well-organized compared with those of PP^+^ MLN-ectomized OVA23-3 mice (Figure S4, Result S4, and Figure 4). Mice devoid of pLT by treatment with LTβR-Ig and TNFR55-Ig fusion proteins were generated as described previously to examine the functions of MLNs and PPs [21]; these mice retain their spleens and isolated lymphoid follicles [20], [21]. When fed the EW diet, pLT^–^ OVA23-3 mice did not develop weight loss or intestinal inflammation (Figure S5 and Result S4). These results indicate that both PPs and MLNs cooperatively promote EW-associated enteropathy in OVA23-3 mice. In addition, splenectomized EW-fed mice showed marked enteropathy similar to that in EW-fed sham-operated mice (Figure 6). In addition, the prevention of inflammation in the EW-fed pLT^–^ OVA23-3 mice, which have spleens and isolated lymphoid follicles only (in the absence of PP and MLNs), supported the dispensable role of the spleen in inflammation. These results confirm that the concurrent absence of both PPs and MLNs led to the lack of clinically apparent food allergy and that the spleen is dispensable in regard to the enteropathy of EW-fed OVA23-3 mice.

## Discussion

Our current study is the first to show the relation between GALT and spleen in the establishment of food-allergic enteropathy in response to orally administered OVA, clarifying the integral roles of MLNs and PPs, but not the spleen. Although the sensitivity of the immune response in each intestinal lymph node population (e.g. pancreatico-duodenal) other than MLNs may influence the ultimate degree of inflammation [30], preparation of MLN-ectomized, PP^–^, PP^–^ MLN-ectomized, and pLT^–^ OVA23-3 mice revealed the necessary coordination between PPs and MLNs in the development of intestinal inflammation. Because the prevention of inflammation was more complete in the absence of both PPs and MLNs than in the absence of either tissue alone, we surmise that PPs contributed to the development of the inflammation by cooperating with MLNs.

Whereas both PP and MLN seem to contribute in generating effector T-cells to establish intestinal inflammation, we posit that their roles are sequential. Specifically, we consider that the responses of PPs are immediate because from results of current study, they are the early and direct inductive sites for orally administered OVA, whereas those of MLNs augment and maintain the PP-induced inflammation thereafter. However the severity of the enteropathy was attenuated in EW-fed PP^+^ MLN-ectomized mice compared with PP^+^ MLN^+^ that is, untreated) EW-fed OVA23-3 mice. In light of the results from CN-fed MLN-ectomized mice, we believe that the surgery itself had no effect on the development of inflammation induced by EW diet feeding in MLN-ectomized OVA23-3 mice. Therefore, although later in the cascade than are PPs, MLNs nonetheless appear to be pivotal in exacerbating the enteropathy and maintaining the associated symptoms.

PPs may not be absolutely required for the development of the enteropathy, as indicated by the blunted response in PP^–^ OVA23-3 mice. However, weight loss and inflammatory responses were exacerbated in control or MLN-ectomized OVA23-3 mice compared with PP^–^ or PP^–^ MLN-ectomized mice, suggesting that PPs provide an important contribution to enteropathy. The morphology of PPs, particularly the interfollicular region, enables the direct activation of CD4^+^ T-cells at this site of OVA uptake by M cells, which overlie PPs and which are involved in food intake [31]. Inflammatory dendritic cells in PPs [9], [32] may facilitate the induction of PP CD4^+^ T-cells to produce the high levels of IL-4 noted on day 10 in EW-fed OVA23-3 mice. The tissues removed from OVA23-3 mice and effects of that removal on the establishment of enteropathy are summarized in Table S1.

The current results also revealed significant differences in the properties of PPs, MLNs, and spleen during the tolerance acquisition phase that followed the enteropathy. Spleen, which is dispensable in regard to intestinal inflammation, appears to contribute to the induction of systemic oral tolerance. On day 28, the induction of systemic tolerance was confirmed by the attenuation of both splenic CD4^+^ T-cell responses (Figure 3A) and serum OVA-specific IgE levels in EW-fed OVA23-3 mice (Figure S2). Concurrent decrease in serum IgE levels with the acquisition of tolerance also occurred in another food allergy model generated through immunization of OVA and alum [33] and in clinical studies of allergen-specific immunotherapy [34]. Even in MLN-ectomized EW-fed OVA23-3 mice, tolerance was induced in splenic CD4^+^ T-cells (Figure S6 and Result S5). On day 10 of the EW diet, although splenic CD4^+^ T-cells were activated and produced IL-4 in response to OVA, the number of CD4^+^ T-cells that infiltrated into the spleen was decreased compared with that in CN-fed OVA23-3 mice. These suggested either T-cell depletion or the induction of apoptosis is a key feature of tolerance induction [35].

In contrast, the induction of tolerance in GALT seemed blunted compared with that in spleen but had a greater effect on PPs than on MLNs in our system. MLNs may be susceptible to excess IL-4 production and thus maintain inflammation in the context of continuous administration of food allergens, even though strong systemic tolerance was induced. In the induction of oral tolerance, the suppression of persistent MLN inflammatory IL-4 responses of effector CD4^+^ T-cells, even after the establishment of systemic tolerance, may have clinical significance in regard to preventing adverse reactions [36], [37]. Distinct from other studies, our current study revealed concurrent differences in the behavior of CD4^+^ T cells between systemic (tolerance) and local (maintenance of priming) tissues in EW-fed OVA23-3 mice. By further improvement, the OVA23-3 mouse model may facilitate delineation of the decisional factors in the blunted T-cell immune responses against oral administered antigens that subsequently cause inflammation versus tolerance. In addition, because antigen-specific T-cells may play an important role in triggering and driving IgE-mediated diarrhea [38], clarifying the mechanism underlying the T-cell mediated intestinal inflammation and developing ways to regulate T-cell responses in food allergy would promote the advancement of specific oral tolerance immunotherapy. Our study further suggests that systemic sensitization by routes other than the intestinal route (e.g., through skin) is needed to induce severe IgE-mediated systemic food-allergy like anaphylaxis [39].

In conclusion, for the effective acquisition of tolerance in food allergy and to improve the induction of tolerance through rational strategies built on clarification of the mechanism, the persistent MLN-associated inflammatory responses must be controlled. However, our study clearly indicates that regulating immune responses in MLNs alone was insufficient, because both PPs and MLNs contribute to the development of the T-cell mediated intestinal inflammation of food allergy. In this regard, PPs are the early and direct inductive sites of immune responses on the intestinal epithelium for uptake of OVA. In addition, PPs reportedly uptake aggregated milk proteins and induce Th2 response [31]. Therefore, regulation of the T-cells in PPs may, through the subsequent attenuation of persistent MLN-driven inflammatory responses, be an easily accessible tool for treating or preventing the exacerbation of intestinal inflammation. An M-cell-targeting delivery system or tolerogenic dendritic cell induction techniques may be valuable in cases involving soluble antigens, such as OVA [40∼42].

## Supporting Information

Figure S1 **The number of or rate of CD4^+^ T-cells in the small intestinal lymphocytes infiltrating into the small intestinal lamina propria of EW-fed OVA23-3 mice during 28 days.**
(TIF)

Figure S2 **Serum concentrations of OVA-specific IgE Abs of EW-fed normal OVA23-3 mice.**
(TIF)

Figure S3 **OVA uptake in PP^−^ OVA23-3 mice.**
(TIF)

Figure S4 **PPs and MLNs cooperatively induce enteropathy in EW-fed OVA23-3 mice.**
(TIF)

Figure S5 **Inhibition of organogenesis in both PPs and MLNs obstructs enteropathy in EW-fed OVA23-3 mice.**
(TIF)

Figure S6 **Proliferation of splenic CD4^+^ T-cells in EW-fed MLN-ectomized OVA23-3 mice.**
(TIF)

Table S1 **Roles of lymphoid tissues in the establishment of enteropathy in EW-fed OVA23-3 mice.**
(TIF)

File S1 **Materials and Methods; Measurement of OVA uptake; Preparation and culture of CD4+ T-cells; Preparation of lamina propria lymphocytes and staining of CD4+ T cells.**
(PDF)

Result S1 **Result of Figure S1; Infiltration of CD4^+^ T cells into the lamina propria of the small intestine of EW-fed OVA23-3 mice.**
(PDF)

Result S2 **Result of Figure S2; Serum concentrations of OVA-specific IgE Abs of EW-fed normal OVA23-3 mice.**
(PDF)

Result S3 **Result of Figure S3; Deficiency of PPs did not influence on ability of OVA uptake in intestinal epithelial cells.**
(PDF)

Result S4 **Result of Figures S4 and S5; Coordinated contribution from PPs and MLNs is crucial to the development of enteropathy in a mouse model of food allergy.**
(PDF)

Result S5 **Result of Figure S6; Proliferation of splenic CD4^+^ T-cells in EW-fed MLN-ectomized OVA23-3 mice.**
(PDF)
