# Supplementary figures and images for: Peyer’s Patches and Mesenteric Lymph Nodes Cooperatively Promote Enteropathy in a Mouse Model of Food Allergy
Source: PLoS One. 2014 Oct 7;9(10):e107492. doi: 10.1371/journal.pone.0107492 (PMC4188560; doi:10.1371/journal.pone.0107492)

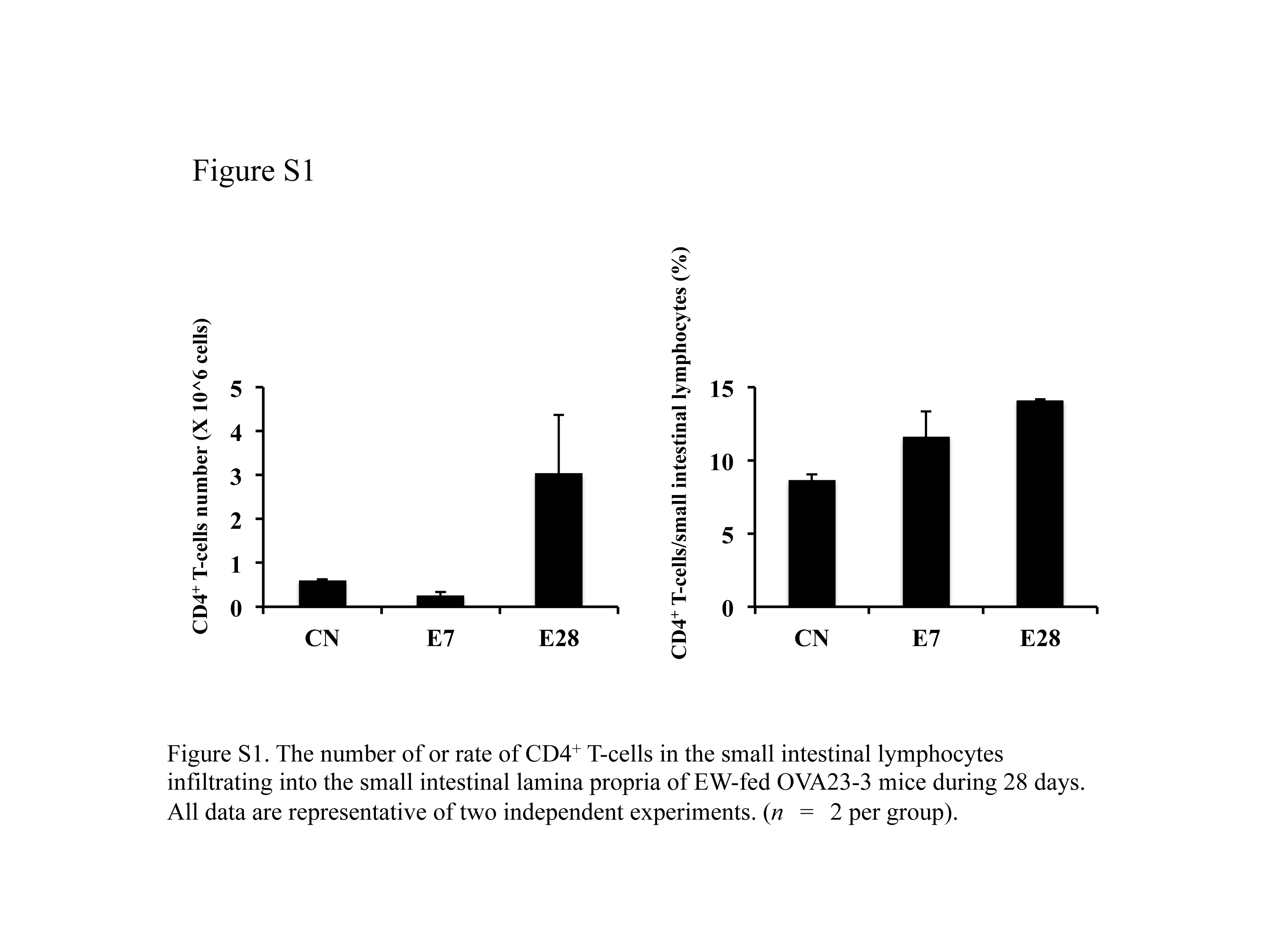

Supplement: Figure S1 — The number of or rate of CD4+ T-cells in the small intestinal lymphocytes infiltrating into the small intestinal lamina propria of EW-fed OVA23-3 mice during 28 days. (TIF) [file pone.0107492.s001.tif]

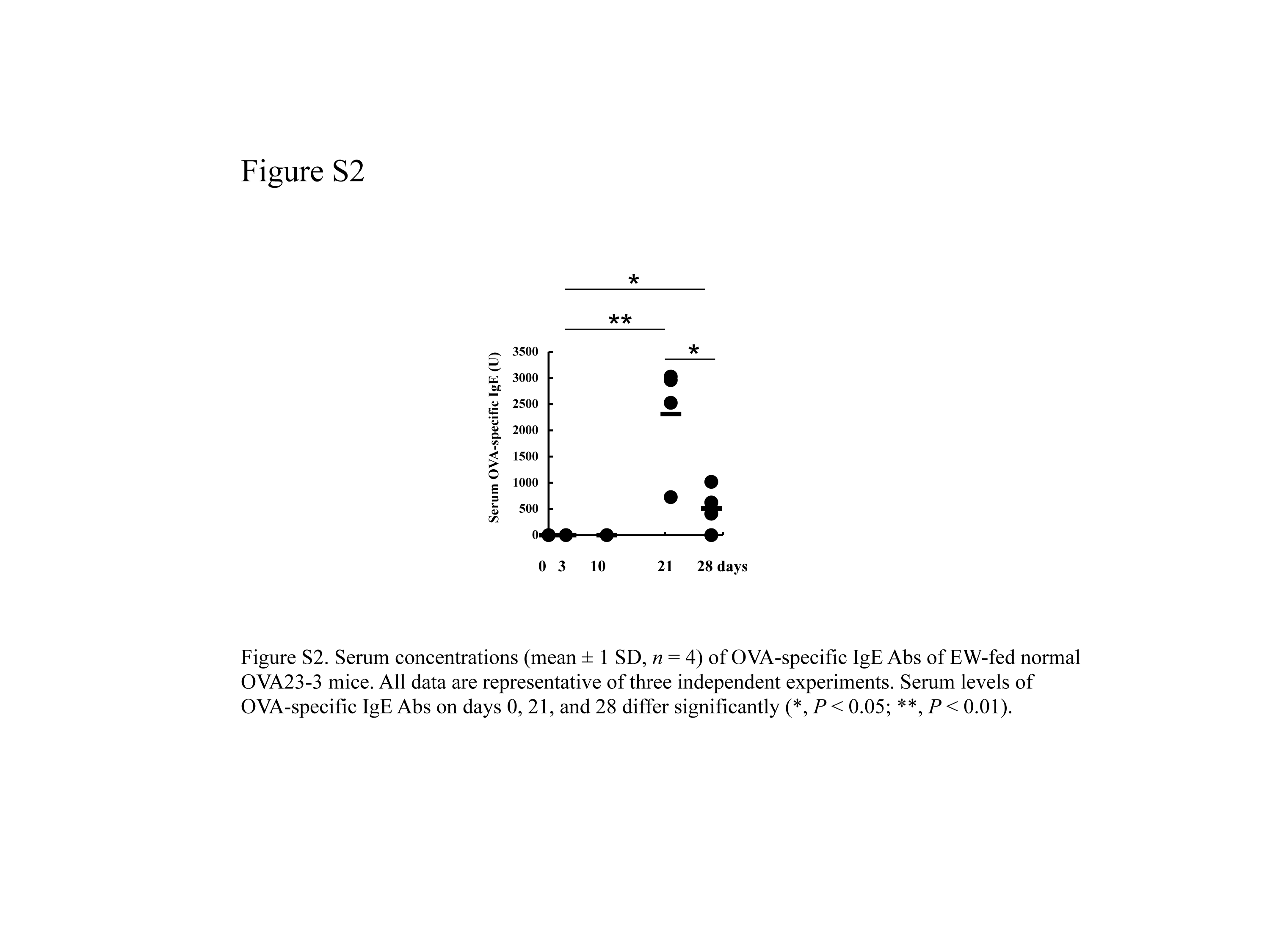

Supplement: Figure S2 — Serum concentrations of OVA-specific IgE Abs of EW-fed normal OVA23-3 mice. (TIF) [file pone.0107492.s002.tif]

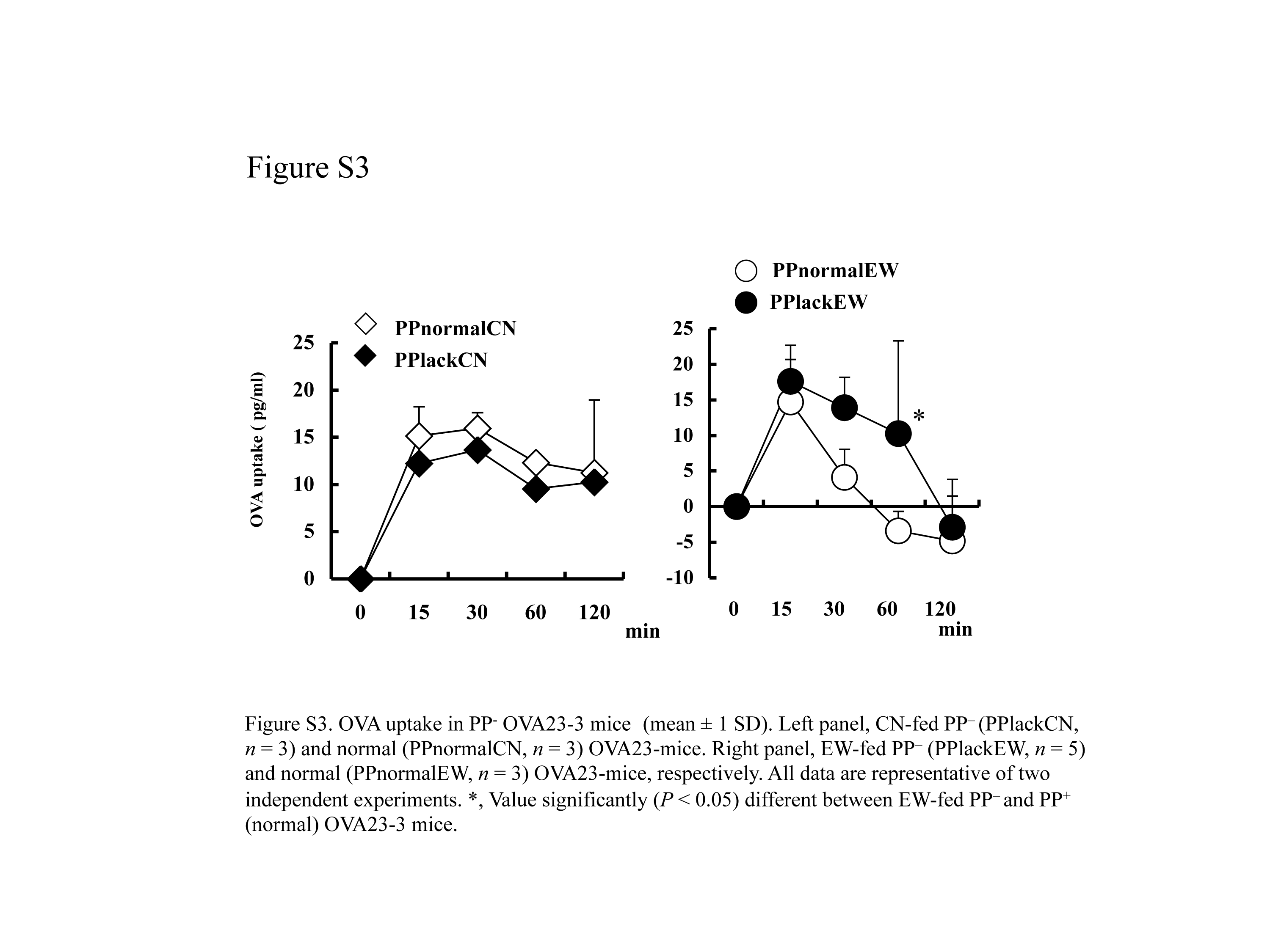

Supplement: Figure S3 — OVA uptake in PP− OVA23-3 mice. (TIF) [file pone.0107492.s003.tif]

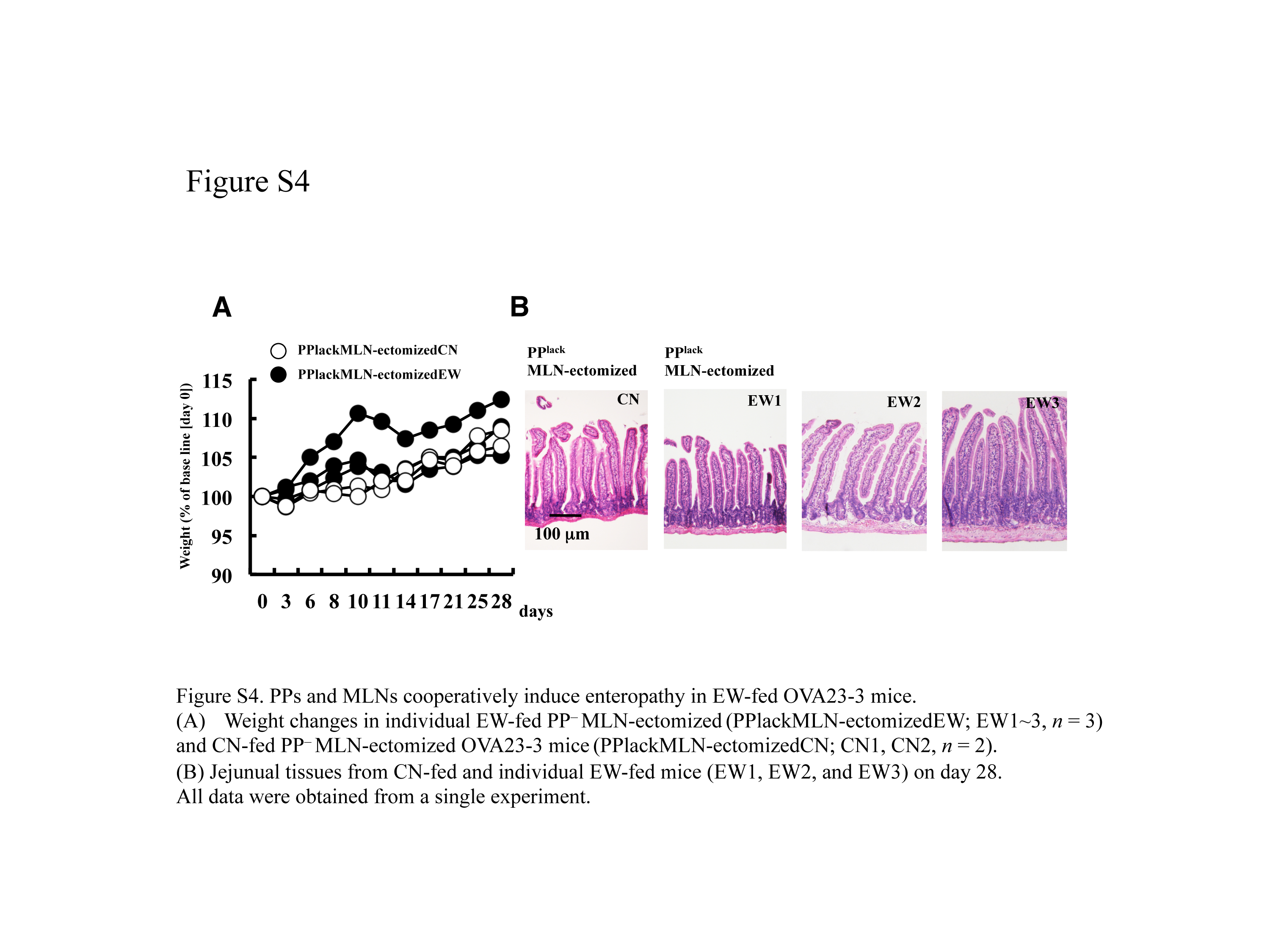

Supplement: Figure S4 — PPs and MLNs cooperatively induce enteropathy in EW-fed OVA23-3 mice. (TIF) [file pone.0107492.s004.tif]

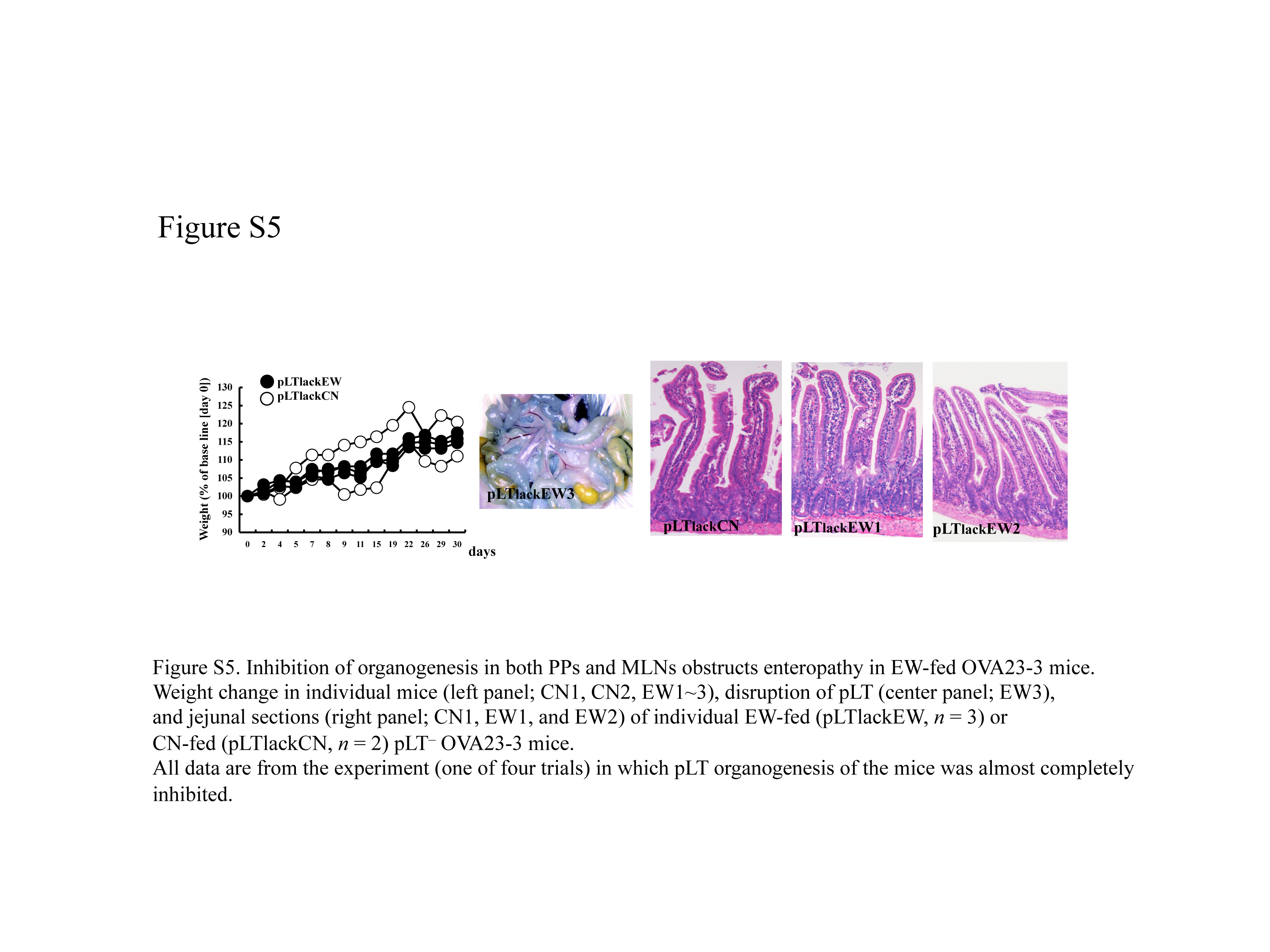

Supplement: Figure S5 — Inhibition of organogenesis in both PPs and MLNs obstructs enteropathy in EW-fed OVA23-3 mice. (TIF) [file pone.0107492.s005.tif]

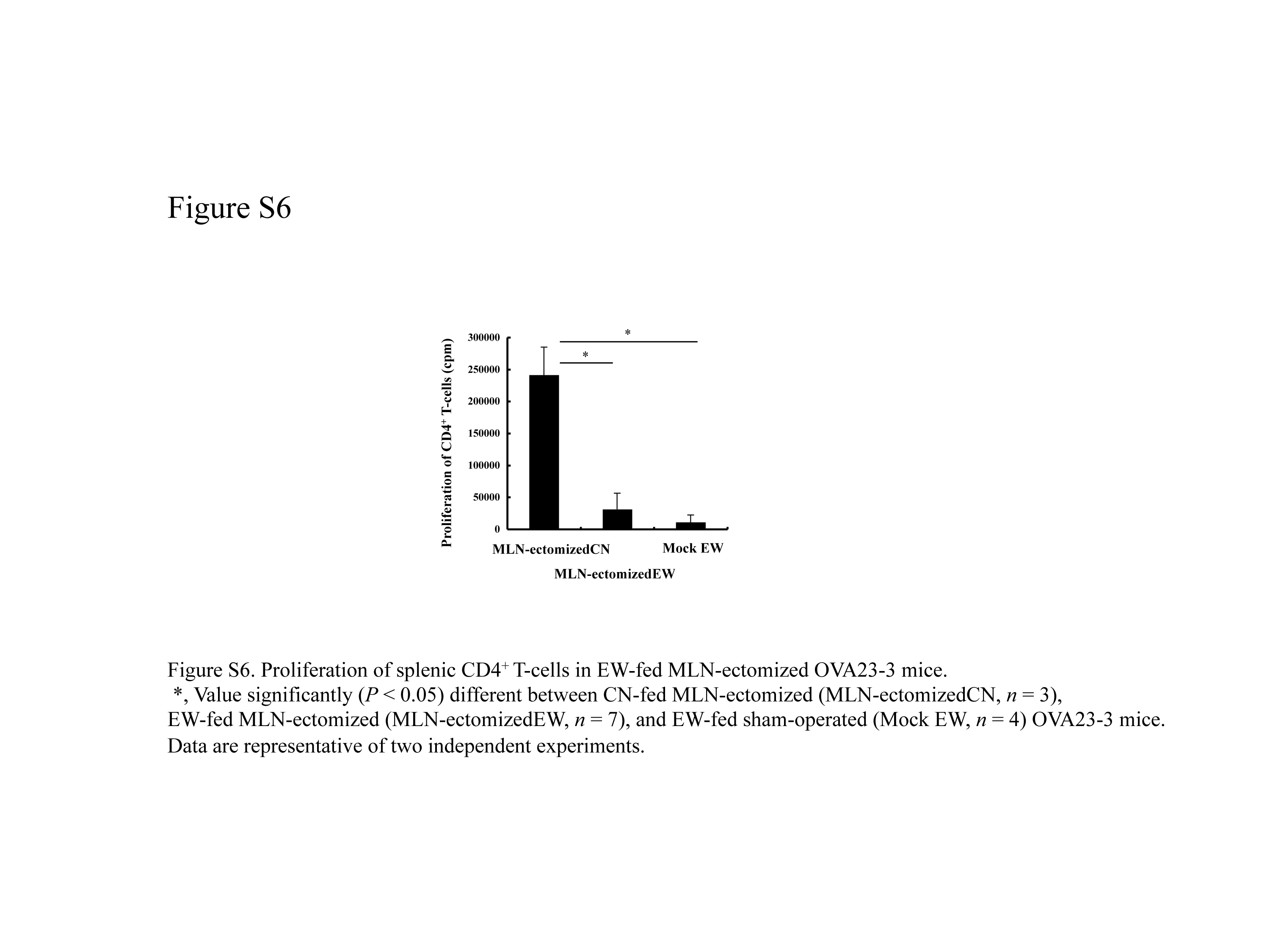

Supplement: Figure S6 — Proliferation of splenic CD4+ T-cells in EW-fed MLN-ectomized OVA23-3 mice. (TIF) [file pone.0107492.s006.tif]

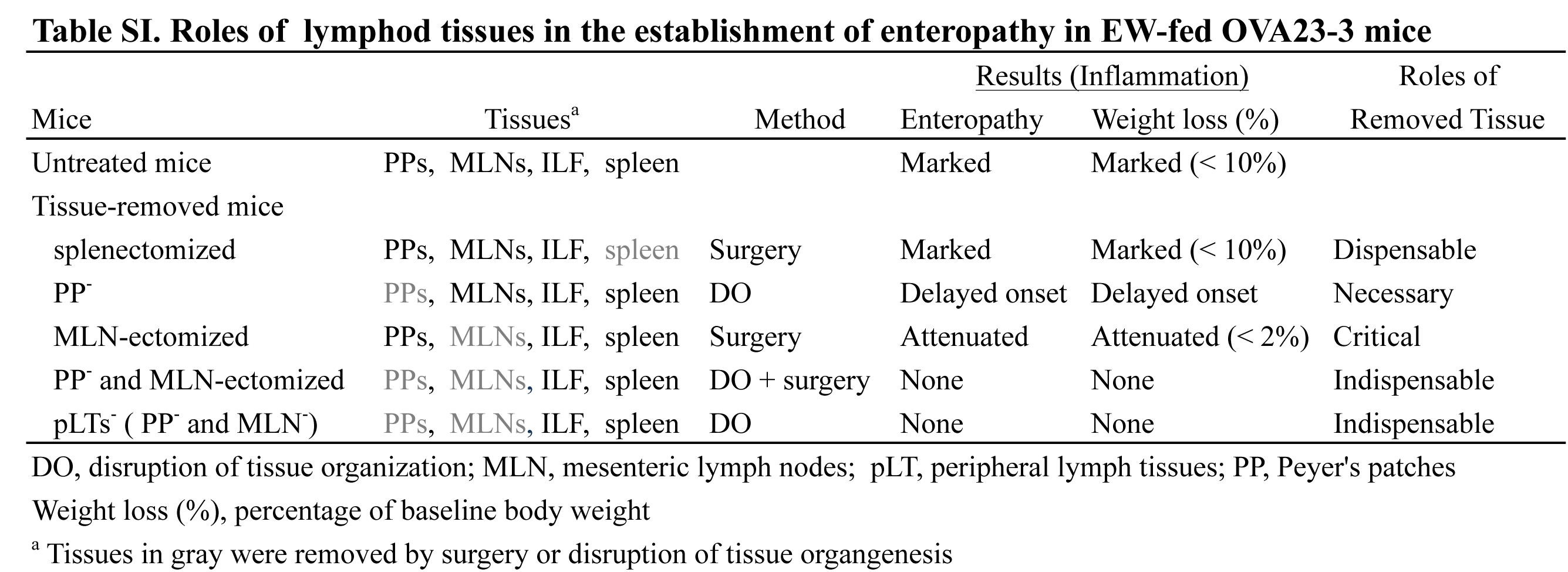

Supplement: Table S1 — Roles of lymphoid tissues in the establishment of enteropathy in EW-fed OVA23-3 mice. (TIF) [file pone.0107492.s007.tif]
