# Supplementary material for: Peyer’s Patches and Mesenteric Lymph Nodes Cooperatively Promote Enteropathy in a Mouse Model of Food Allergy
Source: PLoS One. 2014 Oct 7;9(10):e107492. doi: 10.1371/journal.pone.0107492 (PMC4188560; doi:10.1371/journal.pone.0107492)
Supplement: File S1 — Materials and Methods; Measurement of OVA uptake; Preparation and culture of CD4+ T-cells; Preparation of lamina propria lymphocytes and staining of CD4+ T cells. (PDF) [file pone.0107492.s008.pdf]

Materials and Methods

Measurement of OVA uptake

To analyze OVA uptake on day 3 of feeding the respective diets, EW-fed and CN-fed normal or PP^{-} OVA23-3 mice were food-fasted for at least 3 h before administration of 10 mg OVA (Grade V, Seikagaku Corporation, Tokyo, Japan) diluted in 200 microliter of PBS by orogastric gavage. Blood was collected from their retroorbital plexus at 0, 15, 30, 60 and 120 min after OVA administration; sera were obtained after centrifugation (4℃, 8000 rpm).

Serum concentrations of OVA were determined by ELISA. The wells of 96-well microplates (Maxi Sorp; Nunc, Roskilde, Denmark) were coated at 4°C overnight with 1 mg/ml of rabbit anti-chicken OVA Ab (Bethyl, Montgomery, TX, USA) diluted in PBS. After wells were blocked with 1% rabbit serum in PBS containing 0.05% Tween-20 (PBS-T), each sample and the OVA standard were diluted appropriately with PBS-T containing 1% bovine serum albumin and added to the wells. OVA was detected by using horseradish peroxidase-labeled rabbit anti-chicken OVA Ab. Conjugation of horseradish peroxidase (Roche Diagnostics Japan, Tokyo, Japan) with rabbit anti-chicken OVA Ab (Bethyl, Montgomery, TX, USA) was done by using LC-SMCC (Pierce, Rockford, IL, USA); 3,3’5,5’-Tetramethyl benzidine (Pierce, Rockford, IL, USA) was served as a substrate, and the reaction was stopped with 2N H_2SO_4. The absorbance was measured at 450 nm. Positive reactions were determined by comparison with the concentration of OVA in sera collected before administration.

Preparation and culture of $CD4^+$ T-cells

MLN, PP and splenic $CD4^+$ T-cells were purified from OVA23-3 mice lymphocytes by using a MACS cell-separation system with CD4 beads (Miltenyi Biotec, Bergish Gladbach, Germany). The number and ratio of $CD4^+$ T-cells, which exceeded 95% in each analysis, infiltrating each tissue was determined by staining with an allophycocyanin-conjugated anti-mouse CD4 Ab (GK1.5, Bio legend, San Diego, CA, USA) followed by analysis on an LSR or a FACSCalibur (BD Bioscience, San Jose, CA, USA) flow cytometer. $CD4^+$ T-cells (1×10^5 cells/well) were cultured in the presence or absence of OVA (1 mg/mL) with mitomycin-C treated splenocytes from BALB/cA mice as antigen-presenting cells (4×10^5 cells/well) in complete RPMI1640. After incubation for 48 h, the supernatant was collected from each well to determine cytokine concentration by ELISA, as described previously [12]. To measure T-cell proliferation, $[^3H]$-thymidine (1 microCi; Moravek Biochemicals, Brea, CA, USA) was pulsed into the wells. After incubation for an additional 18 h, $[^3H]$-thymidine incorporation was assayed by liquid scintillation. Proliferation was expressed as the mean number of counts per minute (cpm) of triplicate cultures [12].

Preparation of lamina propria lymphocytes and staining of $CD4^+$ T cells

To examine the infiltration of $CD4^+$ T cells into lamina propria of the small intestine, lamina propria lymphocytes were isolated from CN-fed or EW-fed OVA23-3 mice in accordance with a protocol previously reported [25]. Briefly, after removal of

PPs, the intestine was cut to 2-cm pieces and stirred in RPMI 1640 containing 0.5 mM EDTA and 2% FCS. The tissues then were stirred in 0.5 mg/mL collagenase S1 (Nitta Gelatin, Osaka, Japan) in RPMI 1640 containing 2% FCS. Lymphocytes were isolated at the interface between the 40% and 75% layers of a discontinuous Percoll gradient (GE Healthcare Life Science, Tokyo, Japan), and the total cell number was obtained by staining cells with toluidine blue. Isolated cells were stained with phycoerythrin-conjugated anti-CD4 mAbs (BD Biosciences) and analyzed by using flow cytometry (FACS Calibur, BD Biosciences).
