# Supplementary material for: Peyer’s Patches and Mesenteric Lymph Nodes Cooperatively Promote Enteropathy in a Mouse Model of Food Allergy
Source: PLoS One. 2014 Oct 7;9(10):e107492. doi: 10.1371/journal.pone.0107492 (PMC4188560; doi:10.1371/journal.pone.0107492)
Supplement: Result S1 — Result of Figure S1; Infiltration of CD4+ T cells into the lamina propria of the small intestine of EW-fed OVA23-3 mice. (PDF) [file pone.0107492.s009.pdf]

Result of Figure S1; Infiltration of $CD4^+$ T cells into the lamina propria of the small intestine of EW-fed OVA23-3 mice.

The number of $CD4^+$ T-cells isolated from the small intestinal lamina propria was increased in OVA23-3 mice fed with EW-diet for 28 days compared with CN-fed mice. We were unable to collect enough cells from the lamina propria on day 7 to perform this assessment (Figure S1). The reason for the loss of cells on day 7 remains unknown, although severe inflammation might have affected the recovery of cells from the lamina propria. We also confirmed that the rate of $CD4^+$ T-cells among small intestinal lymphocytes increased throughout the experimental period (Figure S1). These data suggest that EW feeding triggered an influx of $CD4^+$ T-cells to the local inflammatory sites of the small intestine.
