# Supplementary material for: Peyer’s Patches and Mesenteric Lymph Nodes Cooperatively Promote Enteropathy in a Mouse Model of Food Allergy
Source: PLoS One. 2014 Oct 7;9(10):e107492. doi: 10.1371/journal.pone.0107492 (PMC4188560; doi:10.1371/journal.pone.0107492)
Supplement: Result S2 — Result of Figure S2; Serum concentrations of OVA-specific IgE Abs of EW-fed normal OVA23-3 mice. (PDF) [file pone.0107492.s010.pdf]

Result of Figure S2; Serum concentrations of OVA-specific IgE Abs of EW-fed normal OVA23-3 mice.

Serum OVA-specific IgE responses were increased ($P < 0.01$) on day 21 compared with the level before receiving EW diet. On day 28, the IgE responses were significantly ($P < 0.05$) decreased compared with the level on day 21, but still elevated higher ($P < 0.05$) than the level before receiving EW diet (Figure S2).
