# Supplementary material for: Peyer’s Patches and Mesenteric Lymph Nodes Cooperatively Promote Enteropathy in a Mouse Model of Food Allergy
Source: PLoS One. 2014 Oct 7;9(10):e107492. doi: 10.1371/journal.pone.0107492 (PMC4188560; doi:10.1371/journal.pone.0107492)
Supplement: Result S3 — Result of Figure S3; Deficiency of PPs did not influence on ability of OVA uptake in intestinal epithelial cells. (PDF) [file pone.0107492.s011.pdf]

Result of Figure S3; Deficiency of PPs did not influence on ability of OVA uptake in intestinal epithelial cells.

A deficiency of PPs potentially could affect antigen uptake by the intestinal epithelial cells. To examine the influence of PPs deficiency on OVA uptake in OVA23-3 mice, we compared serum OVA concentrations between PP^{-} and normal OVA23-3 mice after oral injection of OVA. OVA uptake (the concentration of each sample after 15, 30, 60 and 120 min from the injection) in OVA23-3 mice fed the CN diet was not influenced by PP deficiency (Figure S3, left). In contrast, the serum concentration of OVA in normal OVA23-3 mice on day 3 of feeding the EW diet tended to be lower than that of EW-fed PP^{-} OVA23-3 mice at 15 and 30 min after the injection of OVA ($P < 0.1$) and was significantly lower ($P < 0.05$) at 60 min (Figure S3, right). These results indicate that the delay of inflammatory responses observed in EW-fed PP^{-} OVA23-3 mice was not caused by a lack of OVA uptake through M cells present in PPs. OVA uptake did not differ between normal and PP^{-} mice, a finding that is substantiated by a report stating that soluble antigen was mainly transported through epithelial cells [6]. In addition, these results regarding OVA uptake in both CN-fed normal and PP^{-} mice confirmed treatment female BALB/c mice with anti-IL-7Ralpha mAb on gestational day 14.5 did not affect the epithelial integrity of the offspring. The earlier onset of inflammatory responses in the epithelial cells of OVA23-3 mice fed with EW for 3 days may have induced the significant decrease of OVA uptake.
