# Supplementary material for: Peyer’s Patches and Mesenteric Lymph Nodes Cooperatively Promote Enteropathy in a Mouse Model of Food Allergy
Source: PLoS One. 2014 Oct 7;9(10):e107492. doi: 10.1371/journal.pone.0107492 (PMC4188560; doi:10.1371/journal.pone.0107492)
Supplement: Result S4 — Result of Figures S4 and S5; Coordinated contribution from PPs and MLNs is crucial to the development of enteropathy in a mouse model of food allergy. (PDF) [file pone.0107492.s012.pdf]

Result of Figures S4 and S5; Coordinated contribution from PPs and MLNs is crucial to the development of enteropathy in a mouse model of food allergy

To determine whether PPs and MLNs cooperate to induce enteropathy, we generated OVA23-3 mice devoid of both PPs and MLNs (PP^{-} MLN-ectomized mice). When fed the EW diet, PP^{-} MLN-ectomized OVA23-3 mice showed no associated weight loss or morphologic changes in the small intestine. In particular, the small intestinal villi of the EW-fed PP^{-} MLN-ectomized OVA23-3 mice were well-organized compared with those of PP^{+} MLN-ectomized OVA23-3 mice (Figure 4 and Figure S4; EW1, EW2, and EW3). When fed the EW diet, pLT^{-} OVA23-3 mice did not develop weight loss (Figure S5; EW1, EW2, and EW3) or intestinal inflammation (Figure S5; EW1 and EW2). We showed the completeness of the inhibition of pLT organogenesis (MLNs and PPs) by presenting a panel of the pLT^{-} heterogeneous OVA23-3 mouse feeding EW diet for 28 days in Figure S5 (center panel; pLTlackEW3).
