# Supplementary material for: Peyer’s Patches and Mesenteric Lymph Nodes Cooperatively Promote Enteropathy in a Mouse Model of Food Allergy
Source: PLoS One. 2014 Oct 7;9(10):e107492. doi: 10.1371/journal.pone.0107492 (PMC4188560; doi:10.1371/journal.pone.0107492)
Supplement: Result S5 — Result of Figure S6; Proliferation of splenic CD4+ T-cells in EW-fed MLN-ectomized OVA23-3 mice. (PDF) [file pone.0107492.s013.pdf]

Result of Figure S6; Proliferation of splenic CD4$^+$ T-cells in EW-fed MLN-ectomized OVA23-3 mice.

The proliferative responses of splenic CD4$^+$ T-cells from MLN-ectomized OVA23-3 mice on day 28 of the EW diet were significantly ($P < 0.05$) attenuated compared with those from CN-fed MLN-ectomized OVA23-3 mice (Figure S6). The proliferative response on day 28 was similar between EW-fed MLN-ectomized and sham-operated mice (Mock EW).
